# Change in Norwegian consumer attitudes towards piglet castration: increased emphasis on animal welfare

**DOI:** 10.1186/s13028-020-00522-6

**Published:** 2020-05-26

**Authors:** Marianne Sødring, Ola Nafstad, Torunn Thauland Håseth

**Affiliations:** Animalia, Norwegian Meat and Poultry Research Centre, Lørenveien 38, 0585 Oslo, Norway

**Keywords:** Animal welfare, Boar taint, Consumer attitudes, Immunocastrates, Piglet castration, Vaccination against boar taint

## Abstract

**Background:**

Male piglets are surgically castrated at a young age primarily to prevent pork meat from being tainted with boar taint, an offensive taste and odor that can be present in uncastrated male pigs. The practice of surgical castration is considered to be both stressful and painful for the piglets, and is therefore under scrutiny due to animal welfare concerns. Rearing of intact males or vaccination against boar taint (immunocastration) are two potential alternatives to surgical castration, but in order to successfully implement either of these alternatives, consumer acceptance of the different methods must be taken into consideration as it will be central for future sales of pork products. A consumer survey mapping Norwegian consumers’ attitudes toward piglet castration was conducted to explore whether the consumers’ position regarding castration has changed since an almost identical study was completed in 2008.

**Results:**

The internet-based survey found that Norwegian consumers are comfortable with the current practice of surgical castration with anesthesia, but also that they are open to the alternative method of vaccination against boar taint. When provided additional information stating that vaccination against boar taint may not be able to reduce boar taint to the levels that castration with anesthesia does, consumer skepticism towards vaccination increased. When evaluating castration methods, animal welfare was the most important influencing factor. Since the original survey from 2008, animal welfare was also the single factor that has increased the most among a set of assessment criteria when purchasing pork products.

**Conclusion:**

Norwegian consumers regard animal welfare as an important factor both when purchasing pork products and when evaluating different methods of castration, and animal welfare as a factor has increased in importance since the initial survey in 2008. Although the current practice of castration using local anesthesia is still widely accepted among consumers, the acceptance of today’s method has declined since the original survey in 2008.

## Background

In most European countries, male piglets are surgically castrated at a young age. This is primarily done to prevent pork meat from being tainted with an offensive taste and odor that can be present in uncastrated male pigs [1]. This unpleasant odor/taste is known as boar taint, and is mainly caused by the accumulation of two compounds, androstenone and skatol, in the pigs’ fat [2–4]. Androstenone is a male pheromone produced in the testis of male pigs during puberty. When a young piglet is castrated, its sexual maturation, and in turn, androstenon production, ceases.

The practice of surgical castration is considered to be both stressful and painful for the piglets, and is therefore under scrutiny due to animal welfare concerns [1, 5–8]. In 2010, on initiative of the European Commission and the Belgian Presidency, representatives from European actors in the pork production chain signed a declaration to abandon surgical castration of pigs from 1. January 2018 [8]; however, this deadline came and went, unmet. In many European countries, male piglets are surgically castrated by the farmer, often either with only analgesics, or without any anesthesia or analgesics at all [9]. In Norway, however, all male piglets, except breeding animals, are surgically castrated by a licensed veterinarian under local anesthesia when they are a few days old, and receive long-acting analgesics during the procedure to reduce post-surgery pain [10]. Norway has what is considered small-scale pig production; in 2018, a total of 1.7 million pigs were slaughtered [11], and recently, the government set a yearly cap of 2100 pigs per farm [12]. The market is highly regulated, and since Norway is almost completely self-sufficient in pork, import is limited by strict tariffs [13]. Requiring a licensed veterinarian to castrate piglets is likely easier to implement in a small-scale pork production system like that of Norway than it would be in most other European countries that operate on a much larger scale, as the veterinary cost would be too large for the pig producer.

Originally, the Norwegian government intended to ban surgical castration of piglets from 2009, but this ban has yet to come into effect, largely due to lack of acceptable alternative solutions for elimination of boar taint. In 2009, the Norwegian Medicines Agency approved the pharmaceutical drug Improvac^®^ for use in Norway, and since 2012, Norwegian pig producers have had the option to select vaccination against boar taint (immunocastration) as an alternative to surgical castration [14]. Another potential alternative solution to surgical castration is the rearing of intact males. However, neither immunocastration nor rearing of intact males are considered to be fully acceptable substitutes for surgical castration as both alternative methods have drawbacks. Rearing of intact males will likely result in more aggression and sexual behavior in the pen, and the risk of boar tainted meat is much higher [1, 15]. Immunocastrates behave as boars prior to the 2nd vaccine injection, and may thus also exhibit more aggressive and sexual behavior [15, 16]. Furthermore, although vaccination against boar taint generally seems to work as intended, in some cases, immunocastrates have been shown to not respond properly to the vaccine, and may therefore exhibit elevated levels of boar taint [17–19]. To successfully implement either of the potential alternatives to surgical castration with anesthesia, consumer acceptance of the different methods must be taken into consideration as it will be central for future sales of pork products.

In 2008, Fredriksen et al. [17] conducted a consumer survey to map the attitudes of the Norwegian consumers toward surgical castration of pigs and some potential alternatives to surgical castration. The survey found that most participants approved the practice of castration using local anesthesia and did not see a need for alternatives. Although there was substantial skepticism towards vaccination against boar taint in the 2008 survey, this alternative to surgical castration was accepted by most Norwegian consumers provided that the Norwegian authorities approved the method [20]. However, much has changed since the 2008-survey: Globally, plant-based alternatives to meat have grown in popularity, a change often supported by environmental, moral or health reasons [21]; In Europe, the European Declaration to abandon castration was signed [8]; and in Norway, the consumers have shown an increased focus on animal welfare in general [22–24]. Therefore, a replica of the 2008-survey was conducted to explore whether the consumer position regarding castration had changed since the original survey in 2008.

## Methods

Consumer attitudes towards castration of pigs were collected via an internet survey administered in November 2016. IPSOS MMI, a market analysis firm, oversaw the survey, and completed the data collection. A total of 1002 persons participated in the survey, and the web panel was made up of a set of randomly chosen men and women over the age of 18. Sampling of participants was weighted by age, gender, educational level and geographical location in order to reflect the Norwegian population demographics. Participation in the survey was voluntary. To participate in the web-panel, consumers were only required to provide an e-mail address, no other personal information was requested. The survey respondents were asked a total of 11 questions (see Additional file 1). First, the participants were asked general questions regarding consumption of pork products and potential influential factors when purchasing pork products, as well as a question to map the respondents’ knowledge on piglet castration. Subsequently, participants were given the following statement explaining the current practice of surgical castration of piglets:

*Norwegian pigs are slaughtered at 4*–*6* *months of age. At this age, male pigs have reached puberty and are called boars or intact males. Meat from these animals may express boar taint. The current practice in Norway is that all male pigs (except breeding animals) are given local anesthesia and castrated by a vet when they are approximately 10* *days old. The anesthesia reduces pain and stress considerably but does not eliminate all pain. In most countries, piglets are still castrated without any anesthesia. This procedure is primarily done to prevent pork meat from being tainted. If the pigs are not castrated, approximately 10*–*20% of the meat will express boar taint. Boar taint is not perceived equally strong by all consumers, but some perceive the taste and taint as very unpleasant. Another reason for castration is to reduce aggressive behavior among the animals during rearing.*

After reading this statement, the consumers were asked to give their opinion of the current practice. Then, another statement was presented, introducing the respondents to vaccination against boar taint:

*An alternative to piglet castration has been developed, so called vaccination against boar taint. The pigs are given two injections with a vaccine that restrains sexual development. The vaccine is not a hormone, but works by making the pig produce antibodies against their own hormones. Consequently, puberty is ceased, and the pig starts behaving like a castrate. The risk of boar taint in the meat is also eliminated. This method was approved in Norway and the European Union in 2009, and approximately 38.000 Norwegian pigs were vaccination against boar taint in 2015. Meat from vaccinated pigs is safe to eat and studies have shown that the eating quality of meat from vaccinated pigs does not differ from that of meat from sows and castrates*.

This was again followed by a question on the acceptability of four methods of castration; surgical castration with anesthesia; castration without anesthesia; vaccinations against boar taint; rearing of intact males. Respondents who answered that surgical castration with anesthesia and/or vaccination against boar taint could not be accepted were asked to give an unaided answer for rejecting the method(s). The survey conducted in 2016 is an accurate replica of the survey completed in 2008 [20]; however, in the 2016 survey, one additional question was added where the participants were provided information regarding past and ongoing research on vaccination against boar taint:

*The questions you have answered so far are an accurate repetition of the questions posed in a similar survey conducted in Norway in 2008. Over the past 10* *years, many international academic communities have researched how to improve on the method of vaccination against boar taint. However, none have yet to succeed in reducing boar taint to the same low levels that are achieved with surgical castration.*

The respondents were then asked to answer the question regarding acceptability of the four methods of castration again, with this new information in mind. The surveys in 2008 and 2016 were the same in order to eliminate the error margin; hence, changes in responses from 2008 to 2016 can be attributed to changing perceptions in the population.

## Results

### Pork consumption and purchasing influence

Of the 1002 web-panel participants, 92% reported that they eat pork products at least once per month. Fifteen percent ate pork 3–5 days a week, 42% ate pork 1–2 days per week, while 28% said they ate pork products 2–3 times per month. Only 6% the respondents ate pork less than once a month, while the remaining 2% never ate pork. Male participants ate pork more frequently than females; 63% of males ate pork at last once per week, while for the female consumers that number was 49%. Furthermore, respondents over 60 years ate pork less frequently than younger respondents.

The most important factor for the consumers when purchasing pork meat, was good taste (91%), followed by easy to prepare (64%) and appealing appearance (61%) (Table 1). Purchasing a product free of additives and a product that has been produced in Norway was also important to the consumers (54% for each factor). Low fat, low cost and animal welfare were on the bottom of the list, with 49%, 48% and 43% respectively. Women placed more emphasis on animal welfare when buying pork than men (51% vs 35%), and animal welfare concerns when purchasing pork products were higher in respondents in the 60+ age bracket than for respondents in the lowest age bracket. The respondents who considered animal welfare to be an important purchasing factor were given a follow-up question where they were asked to provide unaided answers to which factors they deemed to be the most important for pig welfare in Norway. Here, none of the respondents explicitly mentioned castration as a welfare factor. Enough space for the pigs was the number one welfare concern for the consumers.

**Table 1:** Consumer emphasis on different factors when purchasing pork products

How much do the following factors influence you when buying pork products?					
Purchasing factor	All respondents	18–29 years	30–39 years	40–59 years	60+ years
Good taste	91	89	88	92	94
Easy to prepare	64	67	62	64	63
Appealing appearance	61	50	55	63	69
Free of additives	54	35	45	52	75
Produced in Norway	54	41	49	52	68
Low fat	49	46	40	47	56
Low cost	48	64	50	45	37
Animal welfare	43	34	41	42	50

Consumers could choose one of five levels of emphasis (“very important”, “important”, “somewhat important”, “hardly or not at all important”, and “do not purchase/do not know”) to evaluate each purchasing factor. Only the percent of consumers ranking each factor as either “very important” or “important” is presented in the table

### Surgical castration and alternative methods

When asked whether they were aware that almost all male pigs in Norway, except breeding pigs, are castrated when they are small, 60% of the web-panel participants answered that they were unaware. In the lowest age bracket (18–29), only 19% of the respondents were aware of today’s practice of castrating piglets. In contrast, 54% of those over 60 were aware of piglet castration. Most of the respondents that knew about the current practice of surgical castration of piglets hailed from rural areas. Of the 40% of respondents that knew that piglets are castrated when small, 46% said that castration is first and foremost done to avoid unpleasant flavor and/or taste in the pork meat, 10% suggested it was done to ensure calmer animals, while 36% answered that they did not know why pigs are castrated.

**Fig. 1:**
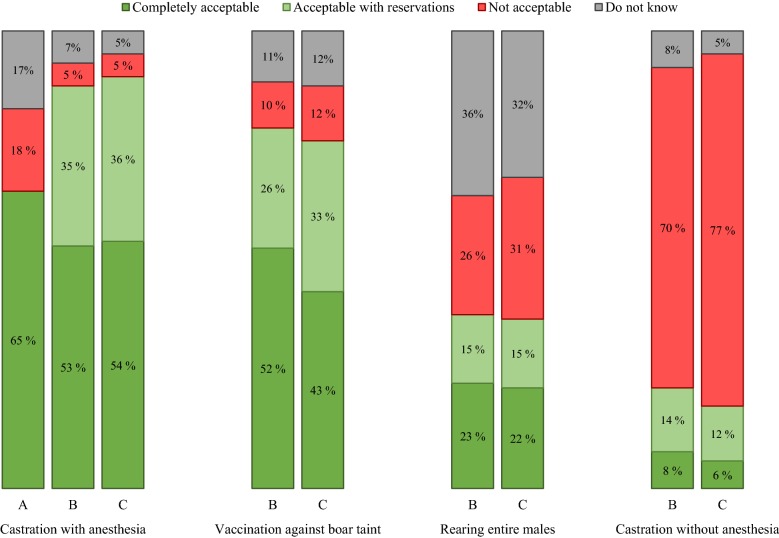
Norwegian consumer attitudes towards the different castration methods. **a** Represents consumer response to surgical castration with anesthesia after being given information on the current practice in Norway. **b** Consumer attitudes towards the four different methods of castration after being informed about vaccination against boar taint, and **c** illustrates consumer attitudes towards the four different methods of castration after receiving additional information regarding the efficacy of vaccination against boar taint

Next, the participants were presented a statement on Norway’s current castration practice and asked to evaluate it. The results showed that 65% of the participants considered the practice to be acceptable, 18% found it unacceptable, while 17% did not have an opinion (Fig. 1). Twice as many women (24%) than men (11%) considered the current practice of surgical castration with anesthesia to be unacceptable.

After completing the survey question evaluating the current castration practice, the web panel was given a statement introducing an alternative to surgical castration; vaccination against boar taint. The respondents were asked to evaluate the current practice of surgical castration with anesthesia up against vaccination against boar taint, surgical castration without anesthesia or rearing of intact males. Both surgical castration with anesthesia and vaccination against boar taint were generally highly accepted among the participants; 88% and 78% respectively, when grouping “completely acceptable” and “acceptable with reservation” for each method (Fig. 1). Of the 1002 respondents, only 5% characterized surgical castration with anesthesia as “not acceptable”, while 10% of the consumers considered vaccination against boar taint to be unacceptable. Younger consumers had a higher preference for vaccination against boar taint than surgical castration with anesthesia; 60% of consumers in the 18–29 age bracket considered vaccination against boar taint to be “completely acceptable” whereas 45% deemed surgical castration with anesthesia “completely acceptable”. For respondents above the age of 60, the contrary was true; 53% considered surgical castration with anesthesia to be “completely acceptable” whilst 43% chose vaccination against boar taint as “completely acceptable”. Female participants were also more opposed to surgical castration with anesthesia than were male respondents. Of the 5% of respondents who considered surgical castration with anesthesia to be unacceptable, 40% stated their answer was based on the information that anesthesia does not completely eliminate all pain for the pig. Other concerns regarding surgical castration with anesthesia included that it is unnatural (11%), unethical (11%), and considered to be a form of animal cruelty (16%). Ten percent of respondents found vaccination against boar taint to be unacceptable, of these, 37% felt that the method was unnatural, while 20% were afraid that the components in the vaccine could potentially affect humans who ate pork from vaccinated pigs. Some respondents (12%) were also concerned about unknown long-term effects when vaccinating against boar taint.

Surgical castration without anesthesia was largely considered unacceptable among Norwegian consumers, with 70% of the respondents choosing this answer (Fig. 1). Of the respondents, as many as 22% considered surgical castration without anesthesia to be acceptable (8% “completely acceptable”; 14% “acceptable with reservation”, Fig. 1). Rearing of intact males was considered an acceptable alternative to the current practice of surgical castration with anesthesia for 38% of the respondents (Fig. 1). When asked which factor they placed more emphasis on when making a choice between the four methods, 73% of the consumers chose animal welfare as important, either as the most important (49%) or equally as important as the consideration for eating quality and food safety (24%) (Table 2). Only 13% and 10%, respectively, chose eating quality and food safety as the most important factor.

**Table 2:** Consumer emphasis on different factors when evaluating the four castration methods: surgical castration with anesthesia; surgical castration without anesthesia; vaccination against boar taint; no castration

When evaluating the four castration methods, what was more important to you? Animal welfare, food safety or eating quality?					
Factor (evaluating methods)	All respondents	18–29 years	30–39 years	40–59 years	60+ years
Animal welfare	49	57	54	51	37
Food safety	10	8	12	11	11
Eating quality	13	9	10	12	19
Equally important	24	20	19	24	28
Uncertain	4	6	4	3	5

Consumers were asked to choose only one of the provided choices when answering the question. All values are presented as percentages

### Additional survey question on castration methods

The respondents were provided an additional statement with information regarding past and ongoing research on vaccination against boar taint, and asked to re-evaluate each of the four methods of castration again. With this new information in mind, overall acceptance for vaccination against boar taint decreased with 2 percentage points (Fig. 1); however, a 9-percentage point decrease was observed for those who chose the answer ‘completely acceptable’, while ‘acceptable with reservations” increased by 7 percentage points. Surgical castration with anesthesia showed an overall acceptance increase of 2 percentage points after the final statement was read.

## Discussion

In 2008, a consumer survey concluded that Norwegian consumers were content with the then current practice of surgical castration with anesthesia and that they accept castration as a necessary means to avoid boar tainted meat [20]. Fredriksen et al. [20] also found that the consumers were skeptical of vaccination against boar taint primarily due to the fear of vaccine residuals in pork meat as well as unknown long-term effects for the consumer. In the present work, a new set of Norwegian consumers were given a questionnaire almost identical to the 2008-survey with the purpose of measuring whether the consumers’ perceptions regarding piglet castration had changed from 2008 to 2016.

### Knowledge of castration

The results of the web-survey revealed that the consumers’ knowledge of piglet castration was as limited in 2016 as it was in 2008; 60% of the respondents were unaware that Norwegian pigs are castrated when young. Other consumer studies have seen comparable trends with low awareness of castration among consumers from Italy, Germany, Belgium, and France [25–29]. The lack of knowledge of current production methods has been suggested to be a result of increasing urbanization and a subsequent disconnect between food production and consumption [30]. It was also clear from the present study that knowledge of piglet castration was lower among younger Norwegians, which is in line with the results seen when the same question was presented in 2008 [20]. This may be due to younger consumers having a more distant relationship to food production than the older generation: Historically, it was more common for Norwegian consumers to have a direct connection to farming and production animals, and consequently, knowledge around production animals is likely higher in older consumers. Moreover, of the respondents in the youngest age bracket, 78% said they lived in an urban area (large city, smaller city), while the remaining respondents lived in a rural setting (small town, countryside). The respondents’ locality may account for the high number of young consumers being unaware of piglet castration.

### Acceptance of alternative castration methods

The present study revealed that Norwegian consumers consider the alternative method of vaccination against boar taint to be almost equally as acceptable as the current castration practice of surgical castration with anesthesia. Previous studies have shown similar high acceptance of vaccination against boar taint for Belgian, French, German, Dutch and Swedish consumers [26, 31]. In contrast, a study with Swiss consumers found that while acceptance for surgical castration with anesthesia was high, acceptance for vaccination against boar taint was low [32]. In the current study, some respondents could not accept vaccination against boar taint as a viable alternative first and foremost because they felt it was unnatural and interfered with nature. They were also skeptical towards the method due to concerns over unknown long-term effects and whether vaccine components could be transferred from pork to human. Likewise, the findings of Mancini et al. [33] mirrors what was observed for Norwegian consumers in the present study; Italian consumers were skeptical towards vaccination against boar taint largely due to fear of potential residues in meat and possible long-term effects on human consumers. Consumer concerns regarding residues is unfounded and likely linked to limited knowledge on vaccination against boar taint. The European Medicines Agency has concluded that the vaccine does not contain harmful residues, and has set the withdrawal time for meat from pigs vaccinated against boar taint at 0 days post slaughter [34].

### Animal welfare concerns

When asked which factors the participants considered to be of importance when purchasing pork, “good taste”, “easy to prepare” and “appealing appearance” were the three leading picks. This mirrors what was observed in the 2008 survey [20]. However, the importance of both “low fat” and “low cost” as purchasing factors has diminished since the 2008 survey [20]. That “low fat” has declined as an important purchasing factor over the past years, may be linked with the fact that Norwegian pork has become leaner since the last survey [35], and has been marketed as such. As in the 2008-survey, the present study revealed that animal welfare had the least impact on consumers when buying pork products. Yet, although animal welfare as a purchasing factor was not ranked as a top priority, this was still the single factor that had increased the most among the purchase assessment criteria from 2008 to 2016. This is in line with the fact that Norwegian consumers’ awareness of, and concern with, animal welfare has increased in the past years [22–24].

Animal welfare is clearly important to consumers; however, at point of purchase, animal welfare often becomes secondary to other criteria. This has been suggested to be due to the consumer choosing to suppress the emotional association to the live animal when buying meat products [30, 36, 37]. This was also observed in the present study, where the consumers ranked the other seven criteria (see Table 1) above animal welfare as influence factors when buying pork products. Animal welfare as a purchase factor was ranked bottom two in all four age brackets, and as the least important for consumers aged 18–29 and 40–59 years. Even so, animal welfare was chosen as the most important factor for evaluating the different castration methods by all respondents regardless of age, indicating that there is a divide between the concept of animal welfare in terms of “live animal” and “food product”. The findings of the present study agree with other, previous Norwegian consumer studies. A large consumer study employing the Citizen jury method to address animal welfare concerns in Norway highlighted that, for Norwegian consumers, ethical viewpoint and consumer behavior is not connected [38]. This was also confirmed in a study where 84% of Norwegian respondents said they had a general interest in animal welfare, yet only 26% thought about animal welfare when buying meat products [39]. Furthermore, a study mapping the Norwegian consumers’ level of knowledge, attitudes towards, and behaviors related to animal welfare, concluded that most consumers appeared to be concerned about the welfare of food production animals and favor animal welfare over price; however, this reflection over animal welfare was absent for most consumers when actually purchasing meat products [40]. This may be due to animal welfare being an ethical matter, leading consumers to unintentionally claim that they give it more emphasis than what is factual and observed at point of purchase [41]. Another reason why animal welfare may not be given more emphasis for Norwegian consumers when purchasing pork products may be that the legislations governing animal welfare in Norway are very strict [10, 42]. According to Skarstad et al. [43] Norwegian consumers simply do not expect to find products associated with poor animal welfare on the Norwegian market, and may thus not feel the need to take this factor into consideration at point of purchase.

Results from the 2016-survey showed that animal welfare concerns again appeared to be of secondary importance when the consumers were informed that boar taint could potentially be present in meat from vaccinated pigs. This shift of emphasis has been observed in multiple studies including a Belgian consumer study where the majority of the respondents were concerned about the welfare of piglets that were surgically castrated without anesthesia; however, although the welfare concern was high, the resolve to pay more for pork from alternative methods was low [28]. When considering animal welfare, Lagerkvist et al. [31] found that Swedish consumers preferred vaccination against boar taint over surgical castration without anesthesia. Yet, when the focus was shifted to boar taint, meat from pigs castrated without anesthesia was actually preferred to meat from intact males, indicating that food quality trumps animal welfare [31].

When providing the reasoning behind the choices made when evaluating the four castration methods, animal welfare was selected as the number one reason by all respondents, and the highest score was found in younger respondents. Interestingly, when evaluating the four methods, but also when asked to give voluntary feedback, the youngest age group clearly emphasizes animal welfare, yet, when asked what influences them most when purchasing pork products, the same age group ranks animal welfare much lower than the other age groups. Notably, “hardly or not at all important” received almost the same percentage score as “very important” and “important” combined, when assessing animal welfare as a purchasing influence factor. Although younger consumers have been found to be more concerned with animal welfare than older consumers, this concern is more geared towards companion animals rather than production animals, and younger consumers may tend to distance themselves from production animals to be able to accept that those animals become food [37, 44]. This may explain the disconnect observed between the scores for animal welfare as a purchasing factor versus as a criterion for castration method evaluation in the younger respondents.

## Conclusions

Norwegian consumers are content with the current practice of castration using local anesthesia, but the acceptance of this method has declined over the past years. Animal welfare has gained more emphasis for consumers, both as a factor when purchasing pork products, but also when evaluating different methods of castration, which is likely the reason Norwegian consumers are more open to alternative methods of castration such as vaccination against boar taint. However, this study also showed that pork quality concerns appear to surpass animal welfare concerns when consumers are informed that vaccination against boar taint may not result in boar taint levels as low as those seen with surgical castration.

## Supplementary information

**Additional file 1.** Consumer survey 2016. Questions included in the consumer survey on different methods of piglet castration.

## Data Availability

The datasets used and/or analyzed during the current study are available from the corresponding author on reasonable request.
