# Supplementary material for: Change in Norwegian consumer attitudes towards piglet castration: increased emphasis on animal welfare
Source: Acta Vet Scand. 2020 May 26;62:22. doi: 10.1186/s13028-020-00522-6 (PMC7249416; doi:10.1186/s13028-020-00522-6)
Supplement: Supplementary file 1 — Additional file 1. Consumer survey 2016. Questions included in the consumer survey on different methods of piglet castration. [file 13028_2020_522_MOESM1_ESM.pdf]

Additional file 1. Questions included in the consumer survey on different methods of piglet castration.

Question	
1	How often do you eat pork products such as ham, pork chops, pork loin, shredded pork, sausages or other pork products?
2	How much do the following factors influence you when buying pork?<br>1. Appealing appearance<br>2. Good taste<br>3. Easy to prepare<br>4. Low cost<br>5. Low fat<br>6. Free of additives<br>7. Animal welfare<br>8. Produced in Norway
3	Which factors do you consider to be the most important for pig welfare in Norway? [a, b]
4	Did you know that almost all male pigs in Norway, except breeding pigs, are castrated when young?
5	Do you know why male pigs in Norway are castrated? [c]
6	What do you think about the current castration practice in Norway?
7	What do you think about each of these four methods?<br>1. Surgical castration with anesthesia<br>2. Surgical castration without anesthesia<br>3. Vaccination against boar taint<br>4. No castration
8	When answering the previous question, what was more important to you? Animal welfare, food safety or eating quality?
9	Why do you consider surgical castration without anesthesia to be unacceptable? [a, d]
10	Why do you consider vaccination against boar taint to be unacceptable? [a, e]
11	What do you think about each of these four methods?<br>1. Surgical castration with anesthesia<br>2. Surgical castration without anesthesia<br>3. Vaccination against boar taint<br>4. No castration

Stippled line indicates where respondents were provided the three statements regarding castration presented in Table 1.

[a] Respondents provided unaided answers to question

[b] Only asked those who considered animal welfare to be rather or very important in question 2 (N = 416)

[c] Only asked those who knew that male pigs are castrated in question 4 (N= 400)

[d] Only asked those who answered that surgical castration with anesthesia was unacceptable in question 7 (N= 54)

[e] Only asked those who answered that vaccination against boar taint was unacceptable question 7 (N= 104)
